# LncRNA DANCR promotes ABL2-mediated metastasis via decoying of miR-125a-5p in high-risk neuroblastoma

**DOI:** 10.3389/fonc.2025.1721248

**Published:** 2026-01-12

**Authors:** Mingyou Gao, Fang Cao, Junling Li, Jun Zhang, Yuren Xia, Xiangdong Tian, Jie Li, Daowei Wang, Lin Lu, Yan Guo, Yuanyuan Liu, Qiang Zhao, Yun Liu

**Affiliations:** 1 Department of Pediatric Oncology, Tianjin Medical University Cancer Institute and Hospital, National Clinical Research Center for Cancer, Tianjin's Clinical Research Center for Cancer, Key Laboratory of Cancer Prevention and Therapy, Department of Genetics, School of Basic Medical Sciences, Tianjin Medical University, Tianjin, China; 2 Department of Thoracic Surgery, The Second Hospital of Tianjin Medical University, Tianjin Medical University, Tianjin, China

**Keywords:** neuroblastoma, DANCR, ABL2, MiR-125a-5p, metastasis, cytoskeleton

## Abstract

**Background:**

Patients with high-risk neuroblastoma preferentially present with widespread metastasis and often relapse despite intensive therapy, which is the major cause of death of patients with this cancer. Consequently, identifying the molecular drivers of neuroblastoma metastasis holds significant clinical importance.

**Methods:**

High-throughput sequencing analysis was performed to evaluate gene expression in neuroblastoma patients. *In vitro* assays were performed to assess cell proliferation, migration, and invasion, while in vivo models were employed to evaluate tumor metastasis. Additionally, integrative transcriptomic and pathway enrichment analyses were utilized to investigate regulatory pathways and molecular mechanisms.

**Results:**

High-throughput sequencing analysis revealed that the long noncoding RNA DANCR was significantly upregulated in high-risk neuroblastoma patients, and its expression correlated with poor prognosis. *In vitro* functional experiments demonstrated that DANCR promotes the proliferation, migration and invasion of neuroblastoma cells. Moreover, DANCR knockdown inhibited tumor metastasis in vivo. Integrative transcriptomic and pathway enrichment analyses further revealed that DANCR overexpression affects the actin cytoskeleton regulatory pathway. Mechanistically, DANCR functions as an oncogenic competing endogenous RNA (ceRNA) through high-affinity binding to miR-125a-5p, thereby promoting ABL2 expression. This DANCR-mediated regulation promotes the interaction between ABL2 and the cytoskeletal regulator cortactin, which leads to activation of the SSH1-cofilin pathway and then facilitates the formation of lamellipodia, cytoskeletal reorganization and the metastatic ability of tumors.

**Conclusion:**

In summary, our findings delineate the DANCR/miR-125a-5p/ABL2/cofilin axis as a critical regulator of cytoskeletal dynamics in neuroblastoma metastasis, offering novel insights for the diagnosis and therapeutic targeting of high-risk neuroblastoma.

## 1 Introduction

Neuroblastoma originates from the sympathetic ganglion or bilateral adrenal gland and is one of the most common extracranial solid tumors in childhood (1). Neuroblastoma is characterized by early onset age and a high frequency of metastasis, and clinical studies have shown that approximately 73% of patients have metastases at the time of diagnosis (2). In high-risk neuroblastoma patients, long-term survival is less than 50%, and relapse survival is less than 10% (3). Despite advances in clinical treatments, including surgery, radiotherapy, chemotherapy and immunotherapy, the prognosis in and mortality of patients with high-risk neuroblastoma have not substantially improved (4, 5). In recent decades, numerous studies have identified a number of important factors affecting prognosis and tumor progression, such as age, MYCN amplification, 11q deletion and tumoral heterogeneity (6). However, metastasis, the main cause of death from this cancer, remains poorly understood (7–9). Therefore, a better understanding of neuroblastoma metastatic mechanisms is essential for improving the prognosis in and treatment of children with neuroblastoma.

Epigenetic regulation, which mainly includes noncoding RNAs, DNA or RNA methylation, histone modifications, and chromosome remodeling, plays a very important role in the development of pediatric tumors, especially neuroblastoma. Long noncoding RNAs (lncRNAs) are a class of RNAs that contain more than 200 nucleotides but are not translated into proteins. LncRNAs are dysregulated in a variety of tumors and are involved in multiple biological processes, including cell proliferation, tumor progression and metastasis (10, 11). In recent years, studies have shown that aberrant expression of lncRNAs plays an important role in neuroblastoma. Through binding to the ribosomal protein RPL35, the long noncoding RNA NB1 induces the translation of the E2F1 protein and activates the transcription of the DEPDC1B gene, which can increase tumorigenesis (12). PRKCQ-AS1 is a superenhancer-driven long noncoding RNA that promotes MYCN-nonamplified neuroblastoma progression by interacting with the MSI2 protein to stabilize BMX mRNA and increase ERK phosphorylation (13). Our previous study demonstrated that the interaction of ADAMTS9-AS2 with LIN28B inhibited LIN28B from interacting with pri-let-7, resulting in the release of pri-let-7 into the cytoplasm and the formation of mature let-7, which in turn inhibited MYCN activity, affecting cancer stemness and differentiation (14). Recently, numerous studies have suggested that lncRNAs can contribute to regulating the biological function or expression of microRNAs (miRNAs) through the competing endogenous RNA (ceRNA) mechanism, which sponges and sequesters miRNAs to modulate specific targets. MicroRNAs can modulate gene silencing by binding to the 3’UTR of lncRNAs or mRNAs, while lncRNAs or mRNAs sharing the same miRNA response element (MRE) can regulate the expression of each other by competitively binding to harbored miRNAs and inhibiting target mRNAs (15, 16). For example, the BRAF pseudogene functions as a ceRNA to upregulate BRAF expression by sequestering shared microRNAs, promoting lymphoma development (17). LncRNA JPX mediates occurrence and metastasis by promoting the Wnt/β-catenin pathway through the miR-33a-5p/Twist1 axis in lung cancer (18). The long noncoding RNA FAM225A can promote oncogenesis and metastasis of nasopharyngeal carcinoma by acting as a ceRNA to sponge miR-590-3p/miR-1275 and upregulate ITGB3 (19). However, the ceRNA mechanism of neuroblastoma metastasis has rarely been elucidated and requires further experimental exploration.

In the present study, on the basis of our previous high-throughput transcriptomic data analysis, we determined that higher expression of differentiation antagonizing nonprotein coding RNA (DANCR) was detected in children with high-risk neuroblastoma than in those with low-risk tumors and was associated with poorer prognoses. DANCR is located at 4q12.5, which is one of the most critical regulatory RNAs in diverse tumors, such as bladder cancer, nasopharyngeal carcinoma, liver cancer, and esophageal cancer (20–24). Further experiments revealed that DANCR could positively regulate the expression of the tyrosine-protein kinase ABL2 by competitively sponging miR-125a-5p, which subsequently stabilizes the actin cytoskeleton to mediate pseudopodia formation and promotes the metastasis ability of neuroblastoma cells. ABL2 and cortactin reportedly interact with each other to stabilize the actin filament and affect the cofilin pathway (25). Furthermore, we confirmed that upregulated DANCR expression significantly promoted the interaction between ABL2 and cortactin, thus activating the SSH1-cofilin pathway. Moreover, regulating the expression of miR-125a-5p significantly reversed the function of DANCR in neuroblastoma. Therefore, our study suggests that DANCR is an oncogenic lncRNA that promotes neuroblastoma progression via the DANCR/miR-125a-5p/ABL2 axis, which may serve as a novel prognostic biomarker and therapeutic target for neuroblastoma.

## 2 Materials and methods

### 2.1 Patients and clinical samples

In the present study, pediatric neuroblastoma samples were obtained from Tianjin Medical University Cancer Institute & Hospital (Tianjin, China). All participants were pathologically diagnosed with neuroblastoma. After the samples were obtained, they were immediately placed in liquid nitrogen for quick freezing and then transferred to -80 °C for storage. The Research Ethics Review Committee of Tianjin Medical University Cancer Hospital approved the study.

### 2.2 Cell culture

Human neuroblastoma cell lines, including SK-N-AS, SK-N-Be2, SH-SY5Y, and HEK293T, were obtained from the American Type Culture Collection (ATCC), while the SK-N-SH and IMR-32 cell lines were sourced from the cell bank of the Chinese Academy of Sciences. The identity of cell lines was authenticated by short tandem repeat analysis. SH-SY5Y, SK-N-AS, and SK-N-SH cells were maintained in Dulbecco’s Modified Eagle Medium (DMEM), enriched with 1% non-essential amino acids. IMR-32 and SK-N-Be2 cells were cultivated in MEM and DMEM/F12, respectively. Each medium formulation was further supplemented with 10% fetal bovine serum (FBS; Gibco, USA) and a combination of 1% penicillin and streptomycin (BI, China). The cells were cultured at 37 °C in a humidified atmosphere of 5% CO_2_. All experiments used early-passage cells and cells were tested for Mycoplasma.

### 2.3 Cell transfection

pcDNA3.1+ was used as the vector for the lncRNA DANCR, and empty pcDNA3.1+ was employed as the negative control. DANCR-siRNA duplexes and nontarget siRNAs were synthesized by GenePharma (China). The plasmid and siRNA sequences are listed in **Supplementary Table S2**. Neuroblastoma cells were transfected with the DANCR plasmid or siRNA using Lipofectamine 2000 Transfection Reagent (Invitrogen, US) following the manufacturer’s instructions.

### 2.4 Isolation of RNA and quantitative real-time PCR

Total RNA was extracted using the RNeasy Mini Kit (QIAGEN, Germany), followed by reverse transcription via either the PrimeScript RT Master Mix (Takara, Japan) or the miRNA First-strand Synthesis Kit (Takara, Japan), following the manufacturers’ protocols. Subsequently, qRT‐PCR was conducted utilizing TB Green Premix Ex Taq II (Takara, Japan). The expression levels of 18S rRNA, GAPDH and U6 served as controls. The qRT‐PCR process was implemented on an Applied Biosystems 7500 Real-Time PCR System. The 2^-ΔΔCt^ method was used to analyze the relative expression of mRNAs/miRNAs. The primers used are listed in **Supplementary Tables S3**, **S4**.

### 2.5 RNA immunoprecipitation

RIP was used to verify the interaction between proteins and RNA as described previously (14). Protein A/G magnetic beads (Thermo, USA) were first incubated with an anti-Ago2 antibody (1:30, Abcam, ab186733) or IgG at 4 °C for 4 h. Neuroblastoma cells were lysed with Pierce IP lysis buffer (Thermo, USA) for 30 min and centrifuged at 12000 rpm for 10 min. The cell lysate and beads were mixed and incubated at 4 °C for 3 hours in the next step. The RNA bound to Ago2 was extracted using the RNeasy Mini Kit (QIAGEN, Germany) and verified by qRT-PCR.

### 2.6 Dual-luciferase reporter assay

Wild-type and mutant pmirGLO dual‐luciferase miRNA target expression vector plasmids (wt-DANCR-miR-125a-5p-luc, mut-DANCR-miR-125a-5p-luc, wt-ABL2-miR-125a-5p-luc, and mut-ABL2-miR-125a-5p-luc) were synthesized by GenePharma (China). The plasmids and the miR-125a-5p mimics or control were cotransfected into SK-N-Be2 and SK-N-AS cells. The dual-luciferase reporter assay system (Promega, USA) was used following the manufacturer’s instructions. First, 1× PLB was added to each culture vessel according to the recommended volume. The culture vessel was shaken for 15 min at room temperature. Thereafter, 100 µl of LAR II was added to 20 µl of lysate to measure firefly luciferase activity. Finally, 100 µl of Stop&Glo Reagent was added to measure Renilla luciferase activity.

### 2.7 Coimmunoprecipitation

For coimmunoprecipitation, cell lysates were incubated on a rotator with antibody. The protein antibody protein A/G-dynabead complexes were prepared. After centrifugation to pelletize the dynabeads, the supernatants were analyzed by SDS-PAGE and immunoblotting, while the precipitated immunocomplexes were subjected to LC-MS/MS or western blot analysis. Co-IP was used to verify the interaction between proteins as described previously (26). The following antibodies were used in Co-IP analysis: Anti-ABL2 (1:50, Abcam, ab134134) and anti-cortactin (1:50, Abcam, ab81208).

### 2.8 Tumor metastasis mouse model

Nude female BALB/c mice aged 5 weeks were used to inject 2 × 10^5^ SK-N-Be2/Scr or SK-N-Be2/shDANCR cells via tail vein (purchased from Beijing Weitonglihua Animal Center, China). The mice were sacrificed by cervical dislocation on 6 weeks after injection. All the animal studies were conducted with approval of the Animal Ethical and Welfare Committee of Tianjin Medical University Cancer Institute and Hospital.

### 2.9 Statistical analyses

The measurement data were compared by Student’s *t* test, ANOVA (with a normal distribution and equal variance), the Mann–Whitney test, and the Kruskal–Wallis test (with a nonnormal distribution or unequal variance). Survival curves were generated using the Kaplan–Meier method and compared using the log-rank test. The overall survival (OS) rate was determined according to the initial diagnosis and death date. KEGG pathway enrichment analysis was conducted using the R package “clusterProfiler”. All the statistical analyses were performed with Prism GraphPad 8. Significant *P* values <0.05, <0.01, <0.001, and <0.0001 are shown as (*), (**), (***), and (****), respectively.

## 3 Results

### 3.1 DANCR is upregulated in high-risk neuroblastoma patients and highly associated with poor prognosis

**Figure 1:**
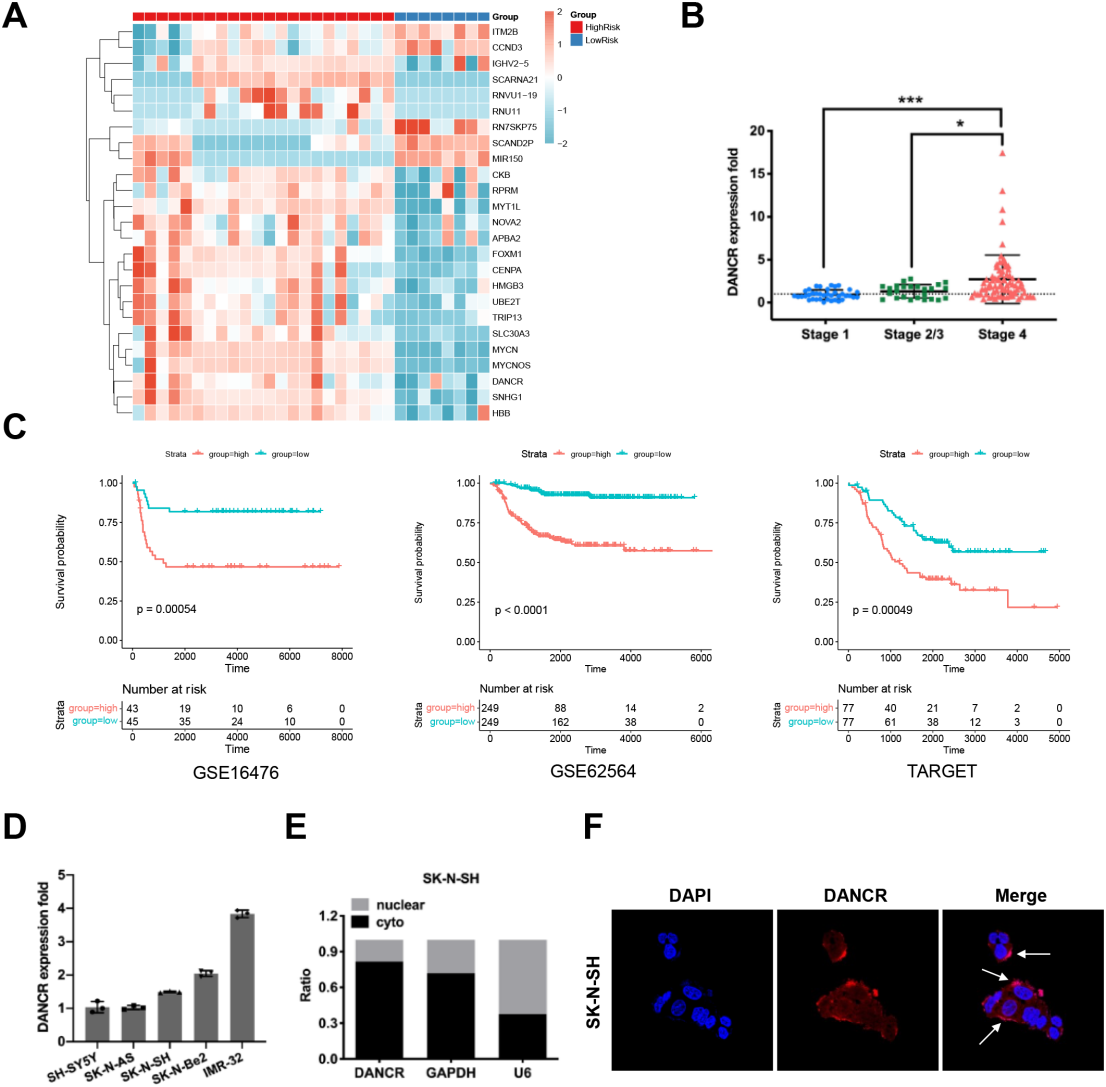
DANCR was overexpressed in the high-risk neuroblastoma specimens and associated with an unfavorable prognosis. **(A)** Transcriptome sequencing showed that DANCR was highly expressed in high-risk neuroblastoma (high-risk 22 cases, and low-risk 8 cases). **(B)** DANCR was measured via qRT-PCR in 139 cases of neuroblastoma tissue. **(C)** Kaplan-Meier curves uncovered that neuroblastoma patients with higher DANCR expression showed poor OS in the database. **(D)** DANCR expression level in five cell lines validated by qRT-PCR: SH-SY5Y, SK-N-AS, SK-N-SH, SK-N-Be2, and IMR-32. **(E, F)** Localization of DANCR in SK-N-SH via Cytoplasmic and Nuclear RNA Purification and RNA FISH.

Firstly, we conducted high-throughput transcriptomic profiling to perform a comparative analysis of the lncRNA expression profiles in neuroblastoma patients at high-risk (22 cases) and low-risk (8cases), all patients were risk-stratified according to the COG (Children’s Oncology Group) neuroblastoma risk grouping classification system, and the results revealed a significant overexpression of the lncRNA DANCR in high-risk neuroblastoma patients (**Figure 1A**, **Supplementary Figure S1A**, **Supplementary Table S1**). Additionally, to investigate the effect of DANCR on neuroblastoma progression, we validated the expression of DANCR at the transcript level in 139 neuroblastoma tissues using qRT-PCR. These findings are consistent with the RNA-sequencing data, which revealed significantly higher expression of DANCR in stage 4 patients than those in earlier stages (**Figure 1B**, P < 0.05). Furthermore, according to the Kaplan-Meier survival analysis conducted with the database (GSE16476, GSE62564, TARGET), neuroblastoma patients with higher expression of DANCR featured a significantly poorer overall survival rate than patients with lower DANCR expression (**Figure 1C**, P < 0.001). To better elucidate the role of DANCR in neuroblastoma cells, we assessed its expression level in five neuroblastoma cell lines (SH-SY5Y, SK-N-AS, SK-N-SH, SK-N-Be2, and IMR-32) using qRT-PCR. As shown in **Figure 1D**, the expression of DANCR was higher in the MYCN amplification cell lines IMR-32 and SK-N-Be2, whereas the expression of DANCR was lower in the SH-SY5Y, SK-N-AS and SK-N-SH cell lines. Moreover, nuclear and cytoplasmic purification and RNA-FISH demonstrated that DANCR is predominantly localized in the cytoplasm (**Figures 1E, F**). Collectively, these results imply that DANCR may have a critical influence on neuroblastoma carcinogenesis.

### 3.2 DANCR promotes proliferation and metastasis in neuroblastoma cells

**Figure 2:**
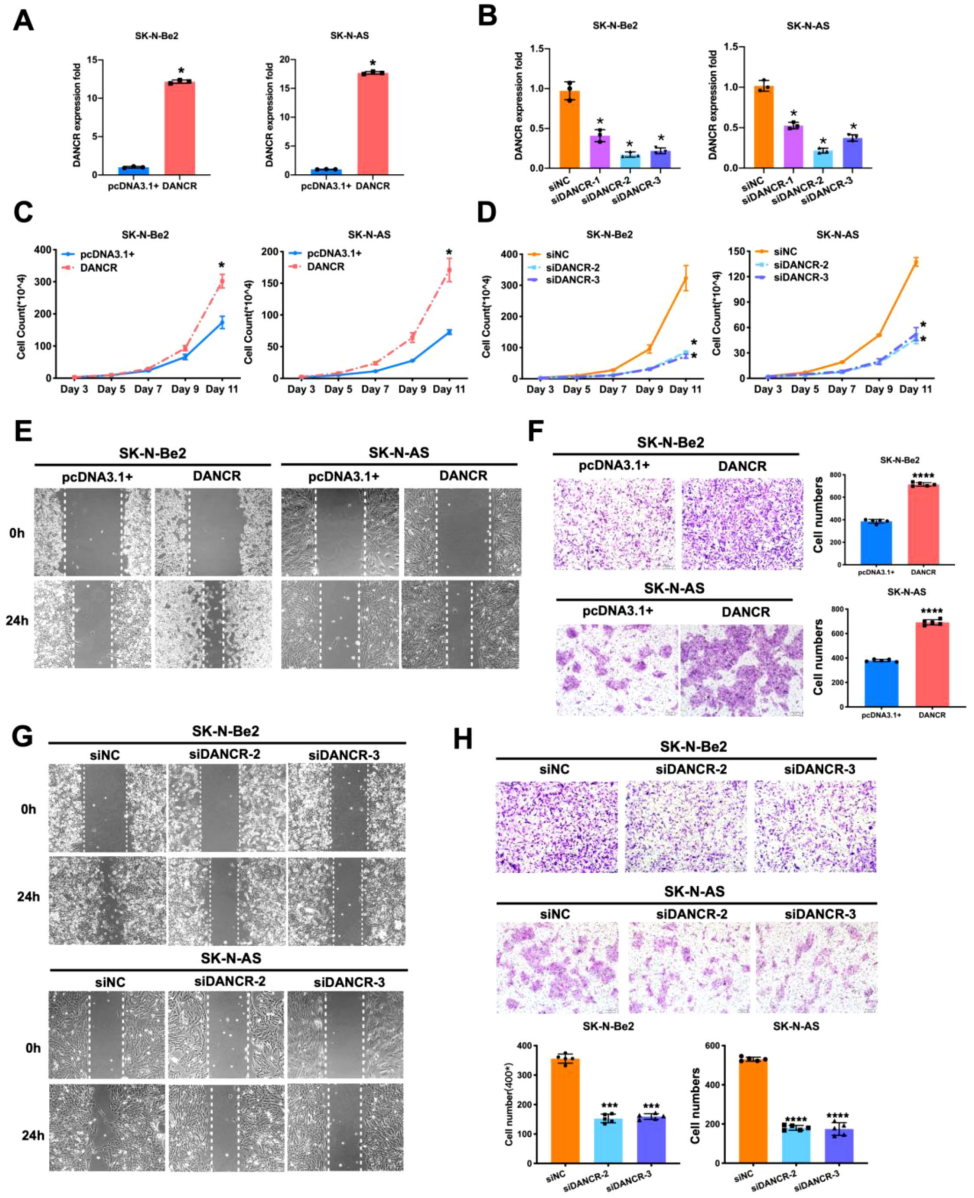
DANCR regulated the proliferation, migration, and invasion of neuroblastoma cells. **(A, B)**. qRT-PCR confirmed the over-expression **(A)** and suppression **(B)** of DANCR in SK-N-Be2 and SK-N-AS cells. **(C, D)** Growth curve assay conducted to determine the cell viability changes after DANCR up- and downregulation in SK-N-Be2 and SK-N-AS cells. E&F. Migration and invasion ability changes after DANCR upregulation in SK-N-Be2 and SK-N-AS cells were determined by wound healing **(E)** and transwell invasion **(F)** assays. G&H. Wound healing **(G)** and Transwell invasion **(H)** assays were utilized to examine the migration and invasion abilities alteration after DANCR downregulation in SK-N-Be2 and SK-N-AS cells. *P < 0.05; ***P < 0.001; ****P < 0.0001.

To elucidate the influence of DANCR on the biological behavior of neuroblastoma cells, we generated the recombinant plasmid pcDNA3.1(+)-DANCR for overexpression and siDANCR for knockdown. We subsequently transfected the SK-N-Be2 and SK-N-AS cell lines, which presented moderate levels of endogenous DANCR expression, as presented in **Figure 1D**. In the neuroblastoma cell lines SK-N-Be2 and SK-N-AS, the pcDNA3.1(+)-DANCR vector significantly induced DANCR overexpression, whereas the siRNA constructs siDANCR-2 and siDANCR-3 effectively inhibited its expression, measured by qRT-PCR (**Figures 2A, B**). We further evaluated the functional effects of DANCR on neuroblastoma cells *in vitro*. Cell growth curve analysis revealed that, compared with the control, DANCR overexpression significantly increased the proliferative ability of SK-N-Be2 and SK-N-AS cells, whereas DANCR knockdown substantially diminished the viability of these neuroblastoma cells (**Figures 2C, D**). In addition, the wound healing assay results indicated that DANCR overexpression promoted the metastatic capacity of SK-N-Be2 and SK-N-AS cells and that DANCR downregulation markedly reduced the migratory distance of these cells (**Figures 2E, G**, **Supplementary Figure S1B**). Consistent with the aforementioned findings, the transwell invasion assay results revealed that the upregulation of DANCR significantly enhanced cell invasion, whereas the silencing of DANCR obviously reduced invasion ability (**Figures 2F, H**). All these outcomes underscore the essential regulatory role of DANCR in the proliferation, metastatic, and invasion capabilities of neuroblastoma cells. In general, DANCR facilitates the malignant phenotype in neuroblastoma.

### 3.3 DANCR is involved in metastasis via the upregulation of ABL2 and triggers SSH1/cofilin signaling in neuroblastoma cells

**Figure 3:**
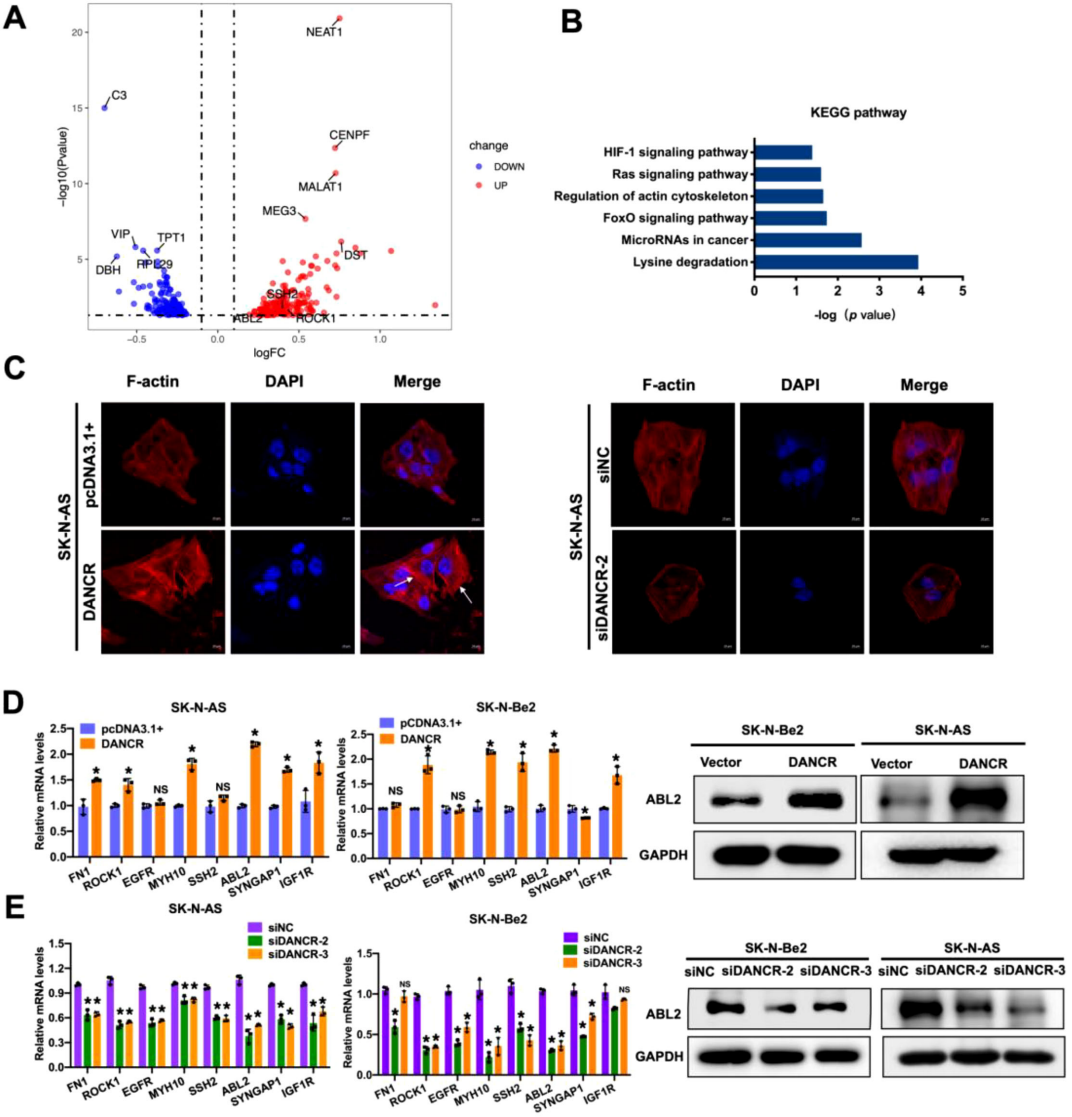
DANCR promoted neuroblastoma metastasis through actin cytoskeleton regulation pathway. **(A)** RNA-sequencing of three pairs of SK-N-AS cells transfected with pcDNA3.1(+)-DANCR and pcDNA3.1(+) empty vector is shown in the volcano plot. **(B)** KEGG pathway enrichment analysis indicated that DANCR was involved in actin cytoskeleton regulation. **(C)** Immunofluorescence images of phalloidin stained SK-N-AS cells with DANCR up- and downregulation. **(D, E)** qRT-PCR verified the expression of cytoskeleton-associated genes after DANCR regulation, and western blotting measured the expression of ABL2. *P < 0.05.

Considering the tumorigenic effect of DANCR in neuroblastoma cells, we aimed to elucidate its underlying molecular mechanism and identify associated regulatory genes. RNA sequencing was performed to determine the differentially expressed genes (DEGs) between the DANCR-overexpressing and empty-vector neuroblastoma cell groups, and the heatmap revealed a significant divergence in the gene expression profiles between the two groups, indicating a substantial impact of DANCR overexpression on the transcriptome landscape (**Figure 3A**). Kyoto Encyclopedia of Genes and Genomes (KEGG) functional enrichment analysis was subsequently performed on these DEGs to delineate the biological pathways potentially involved (**Figure 3B**). Pathway analysis revealed that DANCR is involved in the regulation of the actin cytoskeleton, which is a process integral to the migratory capacity of neuroblastoma cells. Cell migration fundamentally relies on the dynamic rearrangement of the cytoskeleton. As the major component of the cytoskeleton, F-actin plays a crucial role in cell migration by forming lamellipodia, which dynamically extend in the direction of movement through a dynamic process of polymerization and depolymerization (27, 28). Therefore, we investigated the effect of DANCR on the cytoskeleton in neuroblastoma cell lines by immunofluorescence. Indeed, overexpression of DANCR markedly enhanced the formation of lamellipodia, while suppression of DANCR expression substantially depressed lamellipodia formation, suggesting that DANCR could modulate the mobility of neuroblastoma cells by targeting the cytoskeleton (**Figure 3C**).

Given that the overexpression of DANCR could augment the mobility of neuroblastoma cells *in vitro*, we next examined the expression of certain DEGs involved in cell migration and cytoskeleton regulation, including fibronectin 1 (FN1), Rho-associated coiled-coil containing protein kinase 1 (ROCK1), epidermal growth factor receptor (EGFR), myosin heavy chain 10 (MYH10), slingshot protein phosphatase 2 (SSH2), ABL proto-oncogene 2, non-receptor tyrosine kinase (ABL2), synaptic Ras GTPase activating protein 1 (SYNGAP1), and insulin-like growth factor 1 receptor (IGF1R), as identified through KEGG functional enrichment analysis. Among the eight candidate genes, ABL2 showed a significant up-regulation in the DANCR-overexpression group. Furthermore, we confirmed that the expression of these eight genes was relatively elevated in the DANCR overexpression cells compared with the control cells, with the most pronounced increase in ABL2 in the SK-N-Be2 and SK-N-AS cell lines (**Figure 3D**). Consistently, both qRT-PCR and western blotting demonstrated that knockdown of DANCR markedly decreased the expression of these genes, especially ABL2, in SK-N-Be2 and SK-N-AS cells (**Figure 3E**). These findings suggest that ABL2 could serve as an effector gene downstream of DANCR, mediating its influence on neuroblastoma cell behavior.

**Figure 4:**
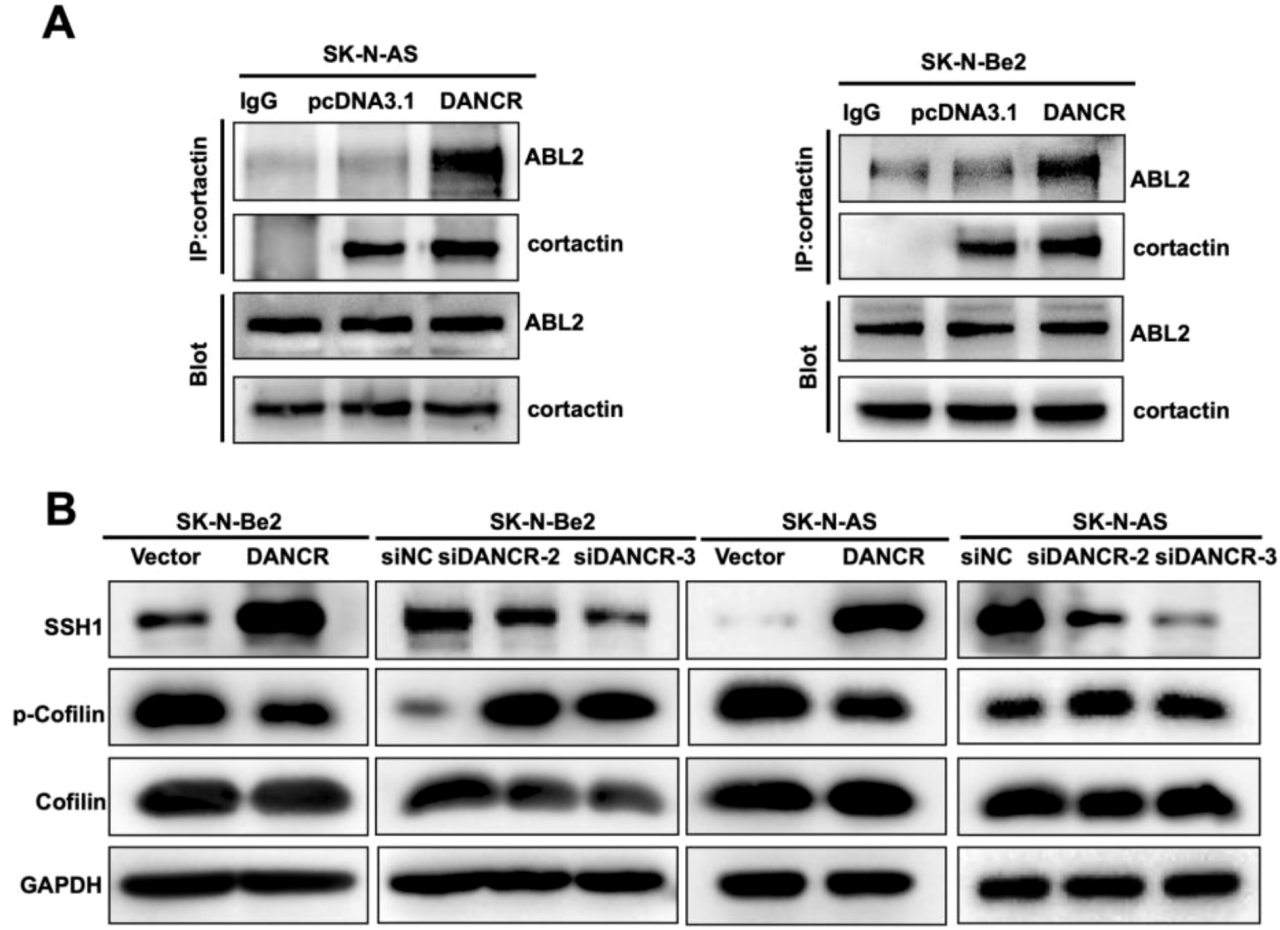
DANCR promoted neuroblastoma metastasis via the ABL2-SSH1-cofilin signal. **(A)** The effect of DANCR expression on the interaction between ABL2 and cortactin was analyzed by co-IP. **(B)** Western blot demonstrated the effect of DANCR on the SSH1-cofilin pathway.

Previous studies have reported that ABL2 regulates the assembly of the action network and modulates the cofilin signaling pathway through its interaction with cortactin (29). Here, we examined the influence of ABL2 on neuroblastoma metastasis. Unexpectedly, the upregulation of ABL2 increased the expression of SSH1 and significantly suppressed the expression of p-cofilin, whereas the overexpression of ABL2 markedly enhanced the formation of lamellipodia (**Supplementary Figures S2A, B**). Therefore, we hypothesized that DANCR may play a role in the cytoskeletal regulatory network, which we verified via coimmunoprecipitation (co-IP) and western blotting in neuroblastoma cells. Co-IP results demonstrated that upregulation of DANCR markedly augmented the interaction between cortactin and ABL2 in SK-N-Be2 and SK-N-AS cells (**Figure 4A**). Additionally, we elucidated the influence of DANCR on the SSH1/cofilin signaling pathway in SK-N-Be2 and SK-N-AS cell lines by immunoblotting. Specifically, the upregulation of DANCR increased the expression of SSH1 but significantly suppressed the expression of p-cofilin, whereas the knockdown of DANCR markedly diminished SSH1 expression and elevated p-cofilin expression. Notably, DANCR had no effect on the total cofilin (**Figure 4B**). Taken together, our findings revealed that DANCR could increase the metastatic ability by activating ABL2 and triggering the SSH1/cofilin signaling pathway.

### 3.4 DANCR upregulated ABL2 via crosstalk with miR-125a-5p

**Figure 5:**
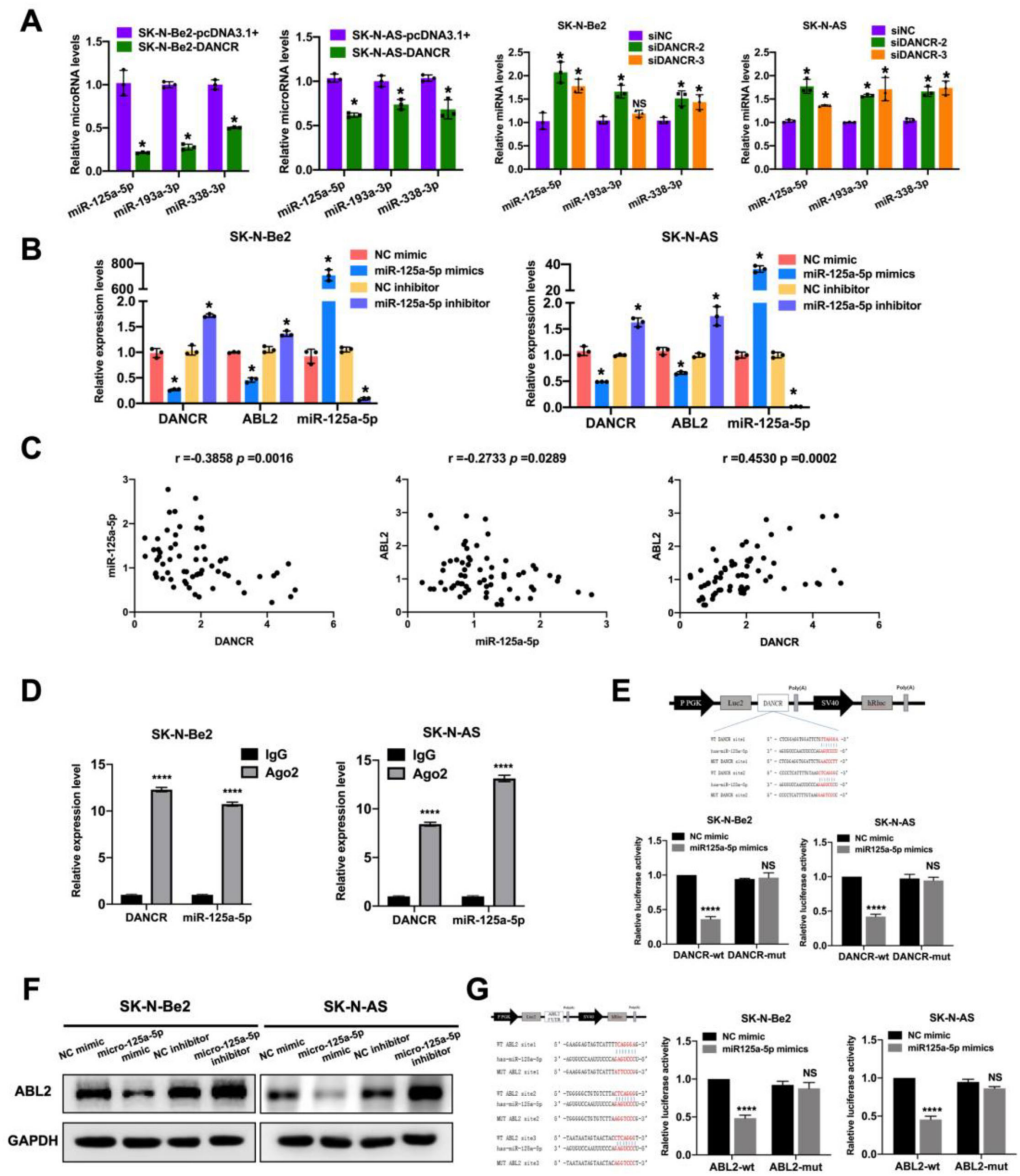
DANCR competitively sponge miR-125a-5p to positively modulate ABL2 expression. **(A)** The expression level of miR-125a-5p, miR-193a-3p, and miR-338-3p in SK-N-Be2 and SK-N-AS cells with DANCR up- and downregulation was tested by qRT-PCR. **(B)** The relationship between DANCR, ABL2, and miR-125a-5p in SK-N-Be2 and SK-N-AS cells was verified using a qPCR assay. **(C)** Pearson correlation analysis confirmed the linkage between DANCR and miR-125a-5p, miR-125a-5p and ABL2, as well as DANCR and ABL2 (n=64). **(D)** RIP and qRT-PCR showed that DANCR and miR-125a-5p were significantly enriched on Ago2 antibody. **(E)** The binding site of DANCR 3′ UTR and miR-125a-5p was proved by a dual-luciferase reporter assay. **(F)** The expression of ABL2 was influenced by the up- and downregulation of miR-125a-5p, as shown by western blotting. **(G)** Dual-luciferase reporter assay indicated the ABL2 3’UTR could bind to miR-125a-5p. *P < 0.05; ****P < 0.0001.

The ceRNA hypothesis demonstrated that lncRNAs can deactivate microRNAs (miRNAs) by sponging them with microRNA response elements (MREs). We indicated that DANCR was positively correlated with ABL2 in the aforementioned sections; therefore, we subsequently investigated the potential miRNAs involved in the DANCR/ABL2 network. The TargetScan (https://www.targetscan.org) and ENCORE (The Encyclopedia of RNA Interactomes, https://starbase.sysu.edu.cn/) databases were utilized to screen downstream miRNA targets, and we discovered that miR-125a-5p, miR-193a-3p, and miR-338-3p could simultaneously bind to DANCR and the 3’ untranslated region (3’UTR) region of ABL2 (**Supplementary Figure S3**). To further validate the relationships among these miRNAs, DANCR and ABL2, we assessed their expression levels in SK-N-Be2 and SK-N-AS cells upon upregulation and downregulation of DANCR by qRT-PCR assays. Our results revealed that all three miRNAs were negatively correlated with the expression of DANCR, with the most pronounced correlation with miR-125a-5p expression. Therefore, we assumed that miR-125a-5p is a candidate miRNA because of its potential regulatory role in the context of DANCR expression (**Figure 5A**). Consistently, qRT-PCR unveiled that elevation of miR-125a-5p by the miR-125a-5p mimic significantly inhibited the expression of both DANCR and ABL2, whereas the knockdown of miR-125a-5p by the miR-125a-5p inhibitor relatively increased DANCR and ABL2 expression in SK-N-Be2 and SK-N-AS cells (**Figure 5B**). The total RNA of 64 neuroblastoma tissue samples was extracted for further validation of the relationships among miR-125a-5p, DANCR, and ABL2. Consistent with previous findings, miR-125a-5p was negatively associated with both ABL2 (r=-0.2733, *P* = 0.0289) and DANCR (r=-0.3858, *P* = 0.0016); nevertheless, ABL2 and DANCR were positively correlated (r=0.4530, *P* = 0.0002; **Figure 5C**). Moreover, the results of the RIP assay utilizing the Ago2 antibody indicated substantial enrichment of DANCR and miR-125a-5p compared with the control group in both SK-N-Be2 and SK-N-AS cells (**Figure 5D**). To validate the binding sites of miR-125a-5p with ABL2 and DANCR, dual-luciferase reporter assays were performed. The cotransfection of wild-type reporter constructs and the miR-125a-5p mimic apparently attenuated the fluorescence intensity of SK-N-Be2 and SK-N-AS cells, whereas the mutant-type reporter constructs had no influence on luciferase activity, confirming the direct interaction between miR-125a-5p and both ABL2 and DANCR (**Figures 5E, G**). In addition, western blot analysis revealed that the miR-125a-5p mimic clearly decreased ABL2 expression, whereas the miR-125a-5p inhibitor moderately increased ABL2 expression (**Figure 5F**). These findings collectively substantiated the hypothesis that DANCR could regulate ABL2 expression via competitively binding with miR-125a-5p.

### 3.5 DANCR decoyed miR-125a-5p to facilitate ABL2/SSH1/cofilin axis mediated metastasis

**Figure 6:**
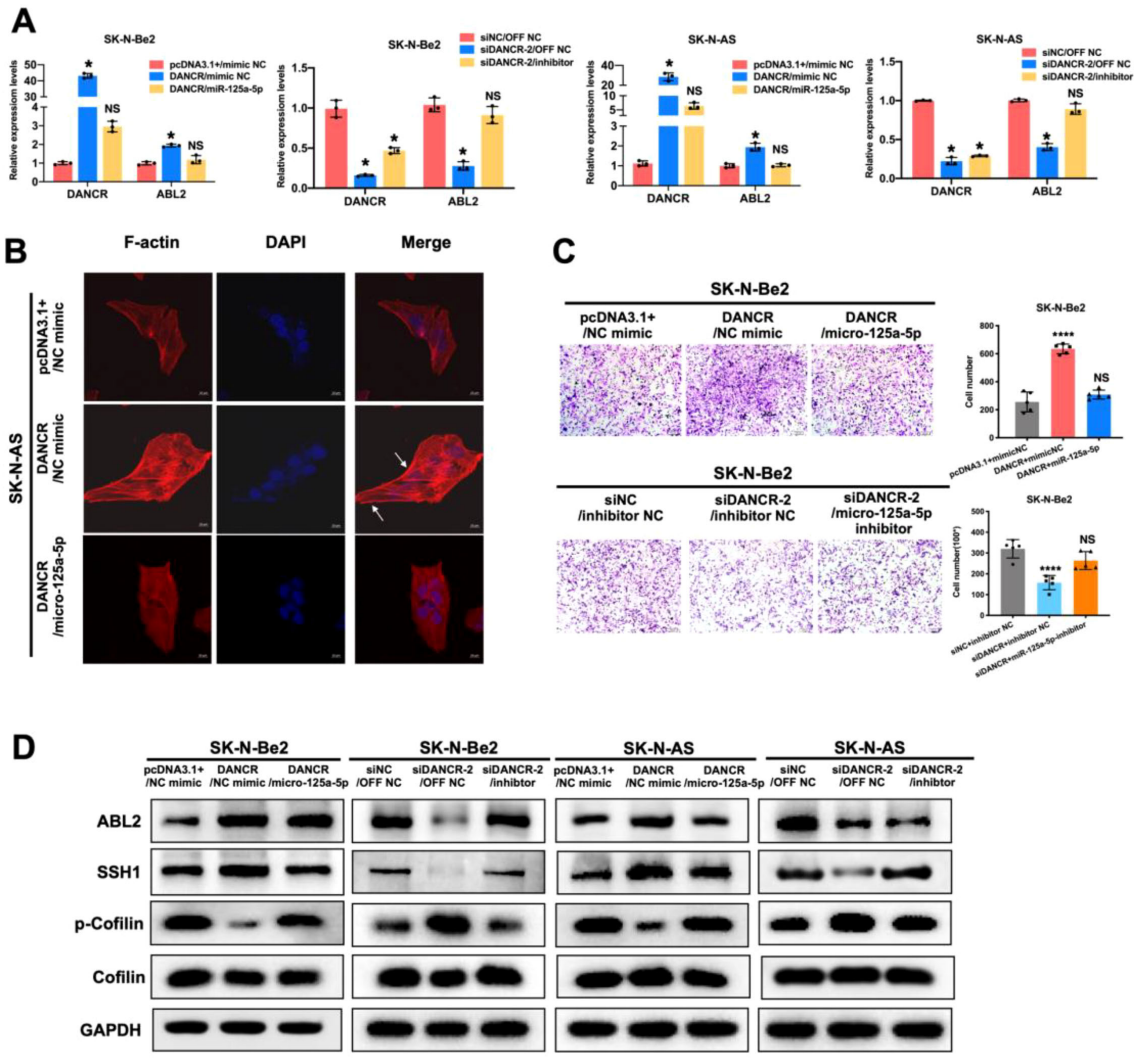
DANCR modulates the metastasis of the neuroblastoma cells by impairing miR-125a-5p-dependent ABL2 inhibition. **(A)** Elevation of miR-125a-5p inhibited ABL2 expression by sponging DANCR as determined by qRT-PCR in SK-N-Be2 and SK-N-AS cells. **(B)** Executed immunofluorescence demonstrated that miR-125a-5p could attenuate DANCR-induced formation of pseudopod in SK-N-AS cells. **(C)** Reperforming the Transwell assay showed that miR-125a-5p profoundly repressed the ability of DANCR in promoting cell invasion in SK-N-Be2 cells. **(D)** miR-125a-5p could reverse ABL2 and SSH1-cofilin pathway protein expression influenced by DANCR in SK-N-Be2 and SK-N-AS cells. *P < 0.05; ****P < 0.0001.

To further confirm the effect of miR-125a-5p on the downstream function of DANCR, we investigated its impact on cellular F-actin organization by immunofluorescence. The results showed that cotransfection of the pcDNA3.1(+)-DANCR construct and the miR-125a-5p mimic markedly impaired ABL2 expression and attenuated the lamellipodium-promoting effect of DANCR (**Figures 6A, B**, **Supplementary Figure S4A**). Uniformly, compared with pcDNA3.1(+)-DANCR/NC mimic transfection, cotransfection of the pcDNA3.1(+)-DANCR construct and the miR-125a-5p mimic significantly reduced the number of cells that crossed the polycarbonate membrane, whereas cotransfection of siDANCR and the miR-125a-5p inhibitor strongly restored the metastatic ability of neuroblastoma cells impeded by siDANCR (**Figures 6A, C**, **Supplementary Figures S4B, C**). Moreover, we found that the miR-125a-5p mimic could impair the elevated protein expression of ABL2 and SSH1 and alleviate the inhibition of p-cofilin, which is induced by DANCR overexpression in both SK-N-Be2 and SK-N-AS cells. Conversely, the miR-125a-5p inhibitor rescued the downregulated protein expression of ABL2 and SSH1 and attenuated the increase in p-cofilin caused by DANCR knockdown (**Figure 6D**). Overall, we concluded that the DANCR/miR-125a-5p/ABL2/SSH1/cofilin signaling pathway regulated the metastatic ability of neuroblastoma.

### 3.6 DANCR promotes metastasis *in vivo*

**Figure 7:**
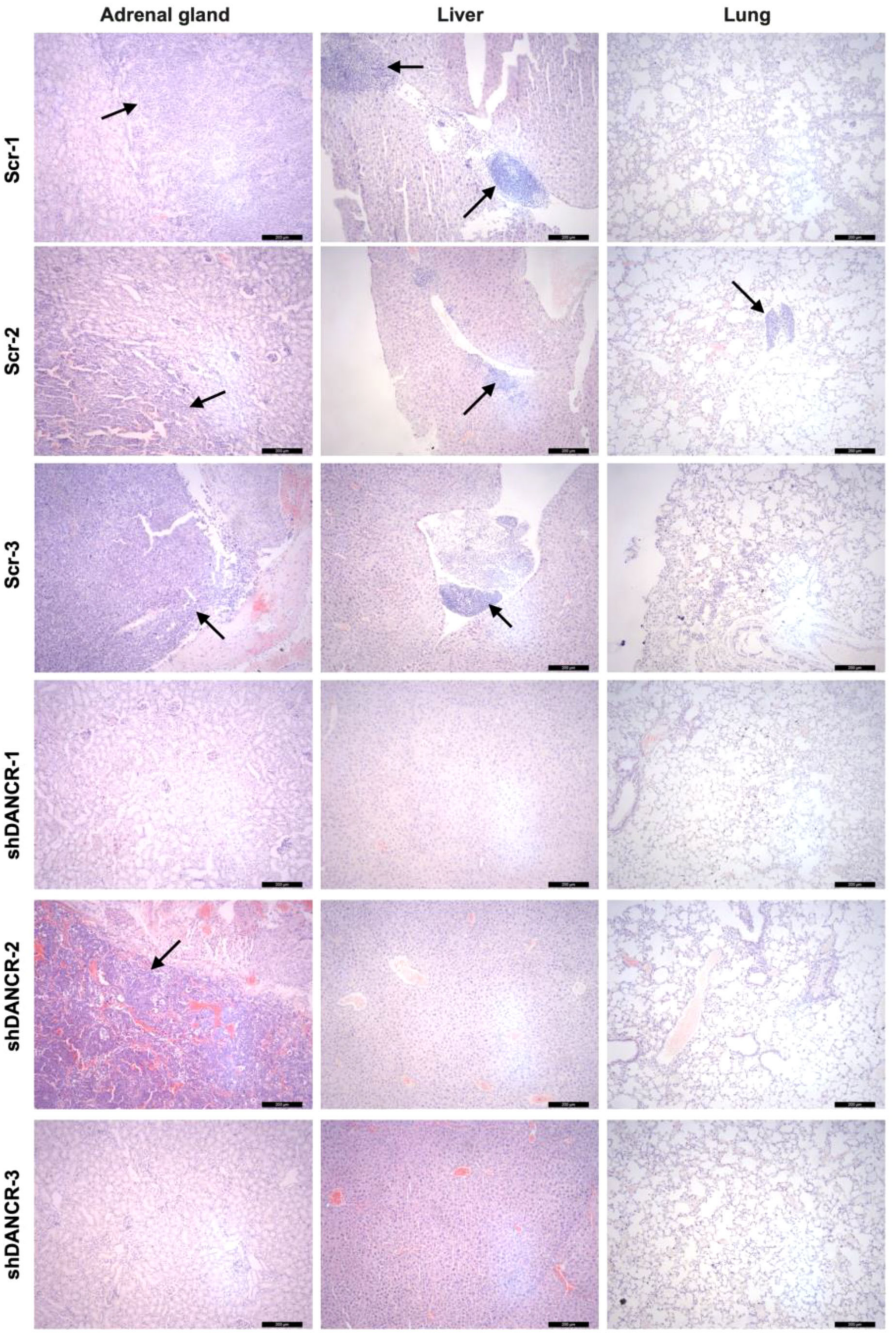
DANCR promotes metastasis *in vivo*. Representative images showing the adrenal gland, liver, and lung metastases (bold arrows) in the metastasis mouse models transplanted with DANCR-knockdown SK-N-Be2 cells and the corresponding control cells. Representative hematoxylin and eosinstained images of the metastatic tumor. Magnification, 100×.

We further examined the role of DANCR in metastasis using a tumor metastasis mouse model. DANCR was stably knocked down in SK-N-Be2 cells (**Supplementary Figure S5**). Metastatic outgrowth in the SK-N-Be2/Scr group was observed in the adrenal glands (3/3, 100%), livers (3/3, 100%), and lungs (1/3, 33%) of the recipient mice at 6 weeks postinjection; the SK-N-Be2/shDANCR group exhibited outgrowth in the adrenal glands (1/3, 33%), livers (0/3, 0%), and lungs (0/3, 0%) (**Figure 7**). DANCR knockdown significantly reduced the metastasis of SK-N-Be2 cells. These results were consistent with the *in vitro* results. Taken together, our results indicate that increased DANCR expression promotes metastasis both *in vitro* and *in vivo*.

## 4 Discussion

Neuroblastoma is the second most prevalent solid tumor in children and is metastatic in 70% of patients at the time of diagnosis (30). The aggressive metastatic potential of neuroblastoma is characterized by its propensity to spread to distant sites, including bone marrow, cortical bone, noncontiguous lymph nodes, and the liver (31). Decades of research on neuroblastoma have uncovered a multitude of factors that contribute to tumor progression and correlate with overall outcome, including patient age; disease stage; histological classification; molecular markers; genetic abnormalities, including MYCN amplification and mutations in genes such as ALK and ATRX; recurrent driver mutations, which are uncommon; and specific alterations associated with neuroblastoma metastasis, which are not fully understood and warrant further investigation. The enhancement of neuroblastoma patient survival necessitates the identification of molecular targets for treatment based on a thorough understanding of the metastatic process. Here, we utilized high-throughput transcriptomic data analysis to identify significant enrichment of DANCR in patients with metastatic high-risk neuroblastoma, which was associated with poorer prognoses.

DANCR has been reported as an oncogene in various carcinomas. Through activating IL11-STAT3 signaling and CCND1, DANCR promoted the metastasis and proliferation of bladder cancer cells (20). DANCR can also function as a ceRNA in osteosarcoma by binding to miR-335-5p and miR-1972, which promote ROCK1-mediated proliferation and metastasis (32). To date, related studies on DANCR and neuroblastoma are rare. As the first encouraging result, we verified that DANCR promoted neuroblastoma cell proliferation and metastasis via the upregulation of ABL2. Enhanced ABL2 expression and activation have been detected in multiple malignancies, including breast, colon, lung, and kidney carcinomas as well as melanoma (33). Notably, ABL2 promotes tumor cell invasion and dissemination while simultaneously suppressing local tumor growth (34). Mechanistically, ABL2 orchestrates cytoskeletal remodeling through multiple pathways: stabilizing F-actin filaments via cortactin binding, which modulates the cofilin-mediated actin depolymerization pathway (25); directly interacting with F-actin through its I/LWEQ motif to assemble actin-rich structures at the cell periphery (35); promoting cortactin phosphorylation and actin polymerization within invadopodia, facilitating extracellular matrix (ECM) degradation and invasive protrusions (36). These processes synergize with F-actin depolymerization and lamellipodia formation, which are key steps in tumor cell migration (37). Crucially, our study links DANCR to ABL2-driven cytoskeletal dynamics in neuroblastoma. We verified that DANCR overexpression in neuroblastoma cells significantly enhances the interaction of ABL2 with cortactin, which also affects SSH1-cofilin activity and stabilizes F-actin networks. This disruption in cytoskeletal regulation induces lamellipodia formation, ultimately accelerating tumor cell invasion and metastasis. Together, these findings establish the DANCR–ABL2 axis as a central regulator of cytoskeletal reorganization and metastatic progression in neuroblastoma, bridging lncRNA function to kinase-driven cytoskeletal plasticity.

LncRNAs take part in several cancer-associated processes, including miRNA silencing, epigenetic regulation, DNA damage, cell cycle control, and signal transduction pathways. In 2011, the ceRNA theory was first proposed by Leonardo Salmena and was extensively accepted in the noncoding RNA area. Numerous studies on ceRNAs and cancers have been conducted (38–40). Distinct miRNAs may directly bind to the 3′ untranslated region of DANCR transcripts, resulting in reduced mRNA stability and protein translation. Specific miRNAs have been implicated in the direct regulation of DANCR expression (41). In our studies, we found that DANCR promoted the metastatic ability of neuroblastoma cells by targeting its downstream gene ABL2. ABL2 has also been broadly reported as a proliferation- and metastasis-related gene in various cancers (33, 34, 42). According to previous studies, ABL2 can be targeted by many miRNAs (43, 44). bMSCs mitigate LPS-induced glycocalyx degradation and vascular leakage by delivering let-7-5p via EVs to target ABL2, suppressing p38MAPK and inflammation (45). Hence, we aimed to elucidate whether DANCR affects ABL2 and ABL2-mediated proliferation and metastasis using a ceRNA mechanism. We therefore wondered whether DANCR could cooperate with miRNAs to regulate ABL2-mediated proliferation and metastasis. We found that DANCR and ABL2 share MREs for miR-125a-5p. Moreover, we revealed a negative correlation between DANCR/ABL2 and miR-125a-5p in neuroblastoma samples by Pearson correlation analysis. MircoRNAs regulate their target genes by directly binding to the complementary seed sequence at the 3′ untranslated region. We designed luciferase and RIP binding assays to confirm the targeted binding effect between DANCR/ABL2 and miR-125a-5p. Convincingly, the constructed luciferase and RIP binding assays confirmed that both DANCR and ABL2 were targets of miR-125a-5p. Furthermore, a series of functional overexpression and knockdown experiment clearly demonstrated the reciprocal suppressive effect between DANCR and miR-125a-5p. Together, we concluded that DANCR promoted ABL2-mediated proliferation and metastasis by acting as a ceRNA by decoying miR-125a-5p in neuroblastoma.

In conclusion, the results of the present study revealed that DANCR functions as a ceRNA to aggravate neuroblastoma metastasis by targeting the miR-125a-5p/ABL2/cofilin axis. Our study highlights the role of DANCR in neuroblastoma metastasis, emphasizes its regulatory relationship with miR-125a-5p and ABL2, and provides a new perspective for neuroblastoma therapy.

## Data Availability

The sequencing data have been deposited in China National Center for Bioinformation under accession number PRJCA006532 (https://ngdc.cncb.ac.cn).
