# Supplementary material for: LncRNA DANCR promotes ABL2-mediated metastasis via decoying of miR-125a-5p in high-risk neuroblastoma
Source: Front Oncol. 2026 Jan 12;15:1721248. doi: 10.3389/fonc.2025.1721248 (PMC12832256; doi:10.3389/fonc.2025.1721248)
Supplement: Supplementary file 1 [file DataSheet1.docx]

**Figure S1** DANCR is upregulated in high-risk neuroblastoma and promotes metastasis. A. DANCR expression in neuroblastoma patients at high-risk (22 cases), and low-risk (8 cases) by LncRNA-sequencing. B. Migration ability changes after DANCR up- and downregulation in SK-N-Be2 and SK-N-AS cells were evaluated using wound healing assay.

**Figure S2** ABL2 promoted neuroblastoma metastasis via the SSH1-cofilin signal. A. Western blotting demonstrated the effect of ABL2 on the SSH1-cofilin pathway. B. Immunofluorescence images of phalloidin stained SK-N-AS cells with ABL2 upregulation.

**Figure S3** DANCR upregulated ABL2 via crosstalk with miR-125-5p. The databases TargetScan (https://www.targetscan.org) and ENCORE (The Encyclopedia of RNA Interactomes, <https://starbase.sysu.edu.cn/>) were applied to predict that miR-125a-5p, miR-193a-3p, and miR-338-3p could simultaneously bind to DANCR and the 3' untranslated region (3'UTR) region of ABL2.

**Figure S4** DANCR decoyed miR-125a-5p to facilitate ABL2/SSH1/cofilin axis-mediated metastasis. A. Cotransfection of pcDNA3.1(+)-DANCR construct and miR-125a-5p mimic. B&C. Cotransfection of pcDNA3.1(+)-DANCR construct and miR-125a-5p mimic significantly impaired the metastatic ability; cotransfection of siDANCR and miR-125a-5p inhibitor restored the metastatic ability of neuroblastoma cells impeded by siDANCR.


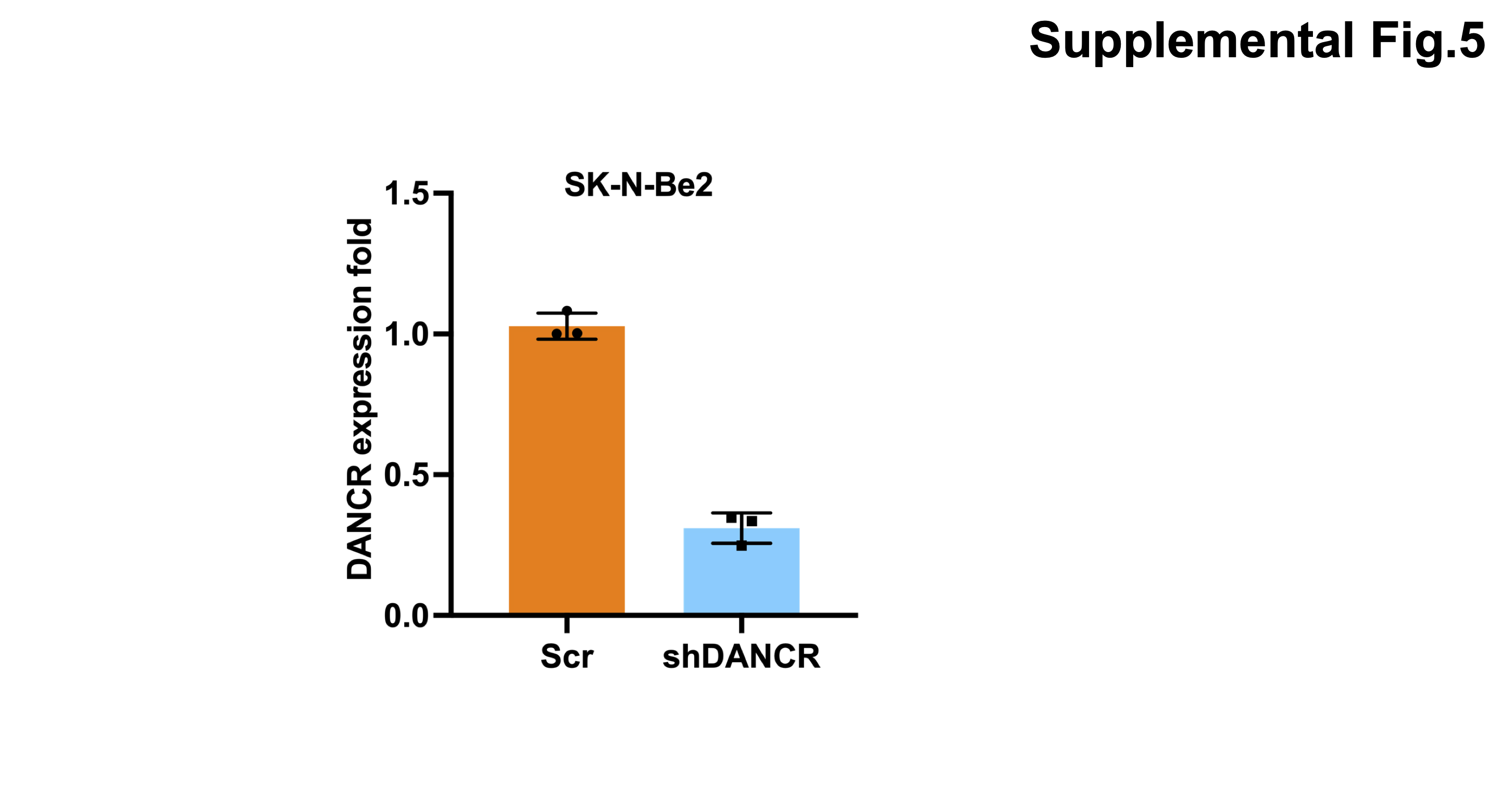


**Figure S5** DANCR promotes metastasis *in vivo*. The mRNA expression levels of DANCR in SK-N-Be2 stable knockdown and control cells were examined using qRT-PCR analysis.
