# Supplementary material for: LncRNA DANCR promotes ABL2-mediated metastasis via decoying of miR-125a-5p in high-risk neuroblastoma
Source: Front Oncol. 2026 Jan 12;15:1721248. doi: 10.3389/fonc.2025.1721248 (PMC12832256; doi:10.3389/fonc.2025.1721248)
Supplement: Supplementary file 2 [file Table1.docx]

**Supplemental tables**

**Table S1 Clinicopathological Information of Neuroblastoma Patients tissue samples for lncRNA-seq**

| Samples | LR1 | LR2 | LR3 | LR4 | LR5 | LR6 | LR7 | LR8 | HR1 | HR2 | HR3 |
| --- | --- | --- | --- | --- | --- | --- | --- | --- | --- | --- | --- |
| Age | ≥18 months | <18 months | ≥18 months | ≥18 months | <18 months | ≥18 months | ≥18 months | ≥18 months | ≥18 months | <18 months | ≥18 months |
| Gender | Female | Female | Male | Male | Female | Female | Female | Female | Male | Female | Female |
| Primary site | Abdomen | thorax | Abdomen | Abdomen | Abdomen | Abdomen | thorax | thorax | Abdomen | Abdomen | Abdomen |
| Metastasis | Absent | Absent | Absent | Absent | Absent | Absent | Absent | Absent | Present | Present | Present |
| MYCN gene amplification | non-amplified | non-amplified | non-amplified | non-amplified | non-amplified | non-amplified | non-amplified | non-amplified | non-amplified | MYCN-amplified | MYCN-amplified |
| Grade of differentiation | maturing subtype | maturing subtype | maturing subtype | maturing subtype | Differentiating | Differentiating | Differentiating | Differentiating | undifferentiated | undifferentiated | undifferentiated |
| INRGSS | L1 | L1 | L1 | L1 | L1 | L1 | L1 | L1 | M | M | M |
| COG Neuroblastoma Risk Grouping | low-risk | low-risk | low-risk | low-risk | low-risk | low-risk | low-risk | low-risk | high-risk | high-risk | high-risk |

**Table S1 Clinicopathological Information of Neuroblastoma Patients tissue samples for lncRNA-seq**

| Samples | HR4 | HR5 | HR6 | HR7 | HR8 | HR9 | HR10 | HR11 | HR12 | HR13 | HR14 |
| --- | --- | --- | --- | --- | --- | --- | --- | --- | --- | --- | --- |
| Age | ≥18 months | ≥18 months | ≥18 months | ≥18 months | ≥18 months | ≥18 months | ≥18 months | ≥18 months | ≥18 months | ≥18 months | ≥18 months |
| Gender | Female | Female | Male | Male | Male | Female | Female | Male | Male | Female | Male |
| Primary site | Abdomen | Abdomen | Abdomen | Abdomen | Abdomen | Abdomen | thorax | Abdomen | Abdomen | Abdomen | Abdomen |
| Metastasis | Present | Present | Present | Present | Present | Present | Present | Present | Present | Present | Present |
| MYCN gene amplification | MYCN-amplified | MYCN-amplified | non-amplified | non-amplified | non-amplified | non-amplified | non-amplified | non-amplified | non-amplified | non-amplified | non-amplified |
| Grade of differentiation | undifferentiated | undifferentiated | undifferentiated | undifferentiated | undifferentiated | undifferentiated | undifferentiated | undifferentiated | Differentiating | undifferentiated | undifferentiated |
| INRGSS | M | M | M | M | M | M | M | M | M | M | M |
| COG Neuroblastoma Risk Grouping | high-risk | high-risk | high-risk | high-risk | high-risk | high-risk | high-risk | high-risk | high-risk | high-risk | high-risk |

**Table S1 Clinicopathological Information of Neuroblastoma Patients tissue samples for lncRNA-seq**

| Samples | HR15 | HR16 | HR17 | HR18 | HR19 | HR20 | HR21 | HR22 |
| --- | --- | --- | --- | --- | --- | --- | --- | --- |
| Age | ≥18 months | <18 months | ≥18 months | ≥18 months | ≥18 months | ≥18 months | ≥18 months | ≥18 months |
| Gender | Male | Female | Male | Male | Female | Male | Male | Female |
| Primary site | thorax | Abdomen | Abdomen | Abdomen | Abdomen | Abdomen | thorax | Abdomen |
| Metastasis | Present | Present | Present | Present | Present | Present | Present | Present |
| MYCN gene amplification | MYCN-amplified | non-amplified | non-amplified | non-amplified | non-amplified | non-amplified | non-amplified | non-amplified |
| Grade of differentiation | undifferentiated | undifferentiated | undifferentiated | undifferentiated | undifferentiated | undifferentiated | undifferentiated | undifferentiated |
| INRGSS | M | M | M | M | M | M | M | M |
| COG Neuroblastoma Risk Grouping | high-risk | high-risk | high-risk | high-risk | high-risk | high-risk | high-risk | high-risk |

Abbreviation: NB, neuroblastoma; INRGSS, International Neuroblastoma Risk Group Staging System; COG, Children’s Oncology Group.

**Table S2. Sequences of the genes coding siRNAs for DANCR knockdown experiments.**

| Name | Target sequence (5’-3’) |
| --- | --- |
| siDANCR-1 | Sense: GUUGACAACUACAGGCACATT  Antisense: UGUGCCUGUAGUUGUCAACTT |
| siDANCR-2 | Sense: CUAGAGCAGUGACAAUGCUTT  Antisense: AGCAUUGUCACUGCUCUAGTT |
| siDANCR-3 | Sense: CUGCAUUCCUGAACCGUUATT  Antisense: UAACGGUUCAGGAAUGCAGTT |

**Table S3. Sequences of the DNA primers for qRT-PCR.**

| Name | Sequence (5’-3’) |
| --- | --- |
| 18S rRNA | Forward: GCTTAATTTGACTCAACACGGGA  Reverse: AGCTATCAATCTGTCAATCCTGTC |
| GAPDH | Forward: TGCACCACCAACTGCTTAGC  Reverse: GGCATGGACTGTGGTCATGAG |
| U6 | Forward: TGGAACGCTTCACGAATTTGCG  Reverse: GGAACGATACAGAGAAGATTAGC |
| DANCR | Forward: GCGCCACTATGTAGCGGGTT  Reverse: TCAATGGCTTGTGCCTGTAGTT |
| ABL2 | Forward: GTTGAACCCCAGGCACTAAAT  Reverse: CAACGAAGAGATTAGGGTCACTC |
| FN1 | Forward: CGGTGGCTGTCAGTCAAAG  Reverse: AAACCTCGGCTTCCTCCATAA |
| ROCK1 | Forward: CACGCCTAACTGACAAGCACCA  Reverse: CAGGTCAACATCTAGCATGGAAC |
| EGFR | Forward: TTGCCGCAAAGTGTGTAACG  Reverse: GTCACCCCTAAATGCCACCG |
| MYH10 | Forward: TCCCGCTGGAGTTTACGC  Reverse: GCAGGAAGCCAAGGAACG |
| SSH2 | Forward: CAAGAATCAGCCACAGACGG  Reverse: TAGGACGATGCTTTCTTCAG |
| SYNGAP1 | Forward: CCTTCAGAGATGTACGGGGAC  Reverse: GTTCCAACCAGGACGATCATAC |
| IGF1R | Forward: TCGACATCCGCAACGACTATC  Reverse: CCAGGGCGTAGTTGTAGAAGAG |

**Table S4. Sequences of the DNA primers for miRNA qPCR.**

| Name | Target sequence (5’-3’) |
| --- | --- |
| hsa-miR-338-3p Forward Primer | UCCAGCAUCAGUGAUUUUGUUG |
| hsa-miR-125a-5p Forward Primer | UCCCUGAGACCCUUUAACCUGUGA |
| hsa-miR-193a-3p Forward Primer | AACUGGCCUACAAAGUCCCAGU |
| U6 | Forward Primer: GGAACGATACAGAGAAGATTAGC  Reverse Primer: TGGAACGCTTCACGAATTTGCG |

**Table S5. KEGG analysis**

| **KEGGID** | **Description** | **GeneRatio** | **BgRatio** | **pvalue** | **geneName** |
| --- | --- | --- | --- | --- | --- |
| hsa00310 | Lysine degradation | 5/50 | 57/5795 | 0.000116435333317875 | KMT2C/KMT2D/KMT2A/KMT2E/NSD1 |
| hsa05206 | MicroRNAs in cancer | 6/50 | 164/5795 | 0.00265636584206358 | EP300/MDM4/ROCK1/EGFR/DICER1/IRS2 |
| hsa04068 | FoxO signaling pathway | 4/50 | 118/5795 | 0.0183101387158992 | EP300/EGFR/IGF1R/IRS2 |
| hsa04810 | Regulation of actin cytoskeleton | 5/50 | 188/5795 | 0.0221578838102637 | FN1/ROCK1/EGFR/MYH10/SSH2 |
| hsa04014 | Ras signaling pathway | 5/50 | 194/5795 | 0.0249905230028045 | SYNGAP1/EGFR/IGF1R/ABL2/AFDN |
| hsa04066 | HIF-1 signaling pathway | 3/50 | 89/5795 | 0.0408845894570121 | EP300/EGFR/IGF1R |
